# Supplementary figures and images for: PAK1-Dependent Regulation of Microtubule Organization and Spindle Migration Is Essential for the Metaphase I–Metaphase II Transition in Porcine Oocytes
Source: Biomolecules. 2024 Feb 17;14(2):237. doi: 10.3390/biom14020237 (PMC10886677; doi:10.3390/biom14020237)

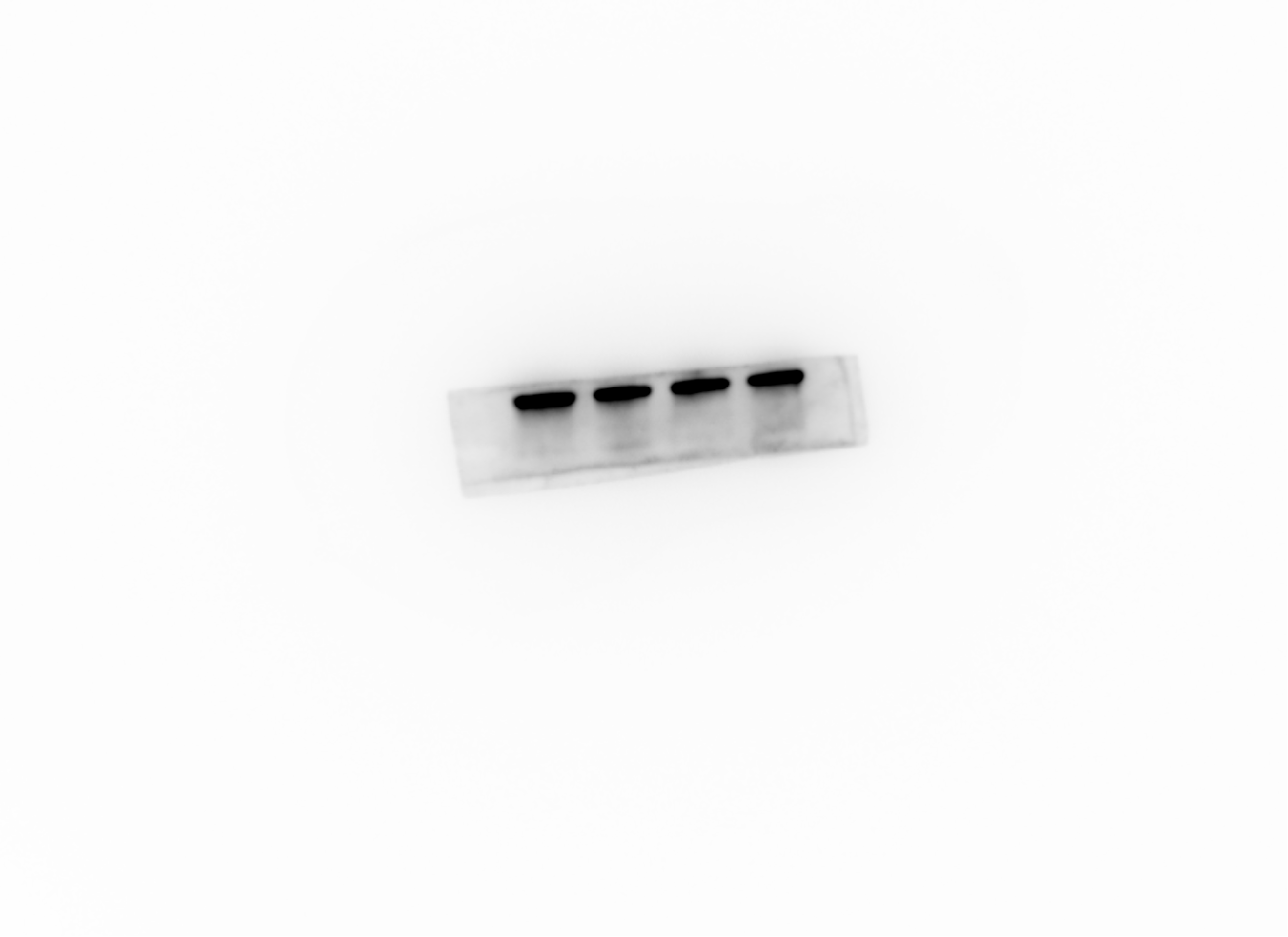

Supplement: Supplementary file 1 [file biomolecules-14-00237-s001.zip › biomolecules-2818843-supplementary/Original images of western Blot/original images/Figure 1A GAPDH.tif]

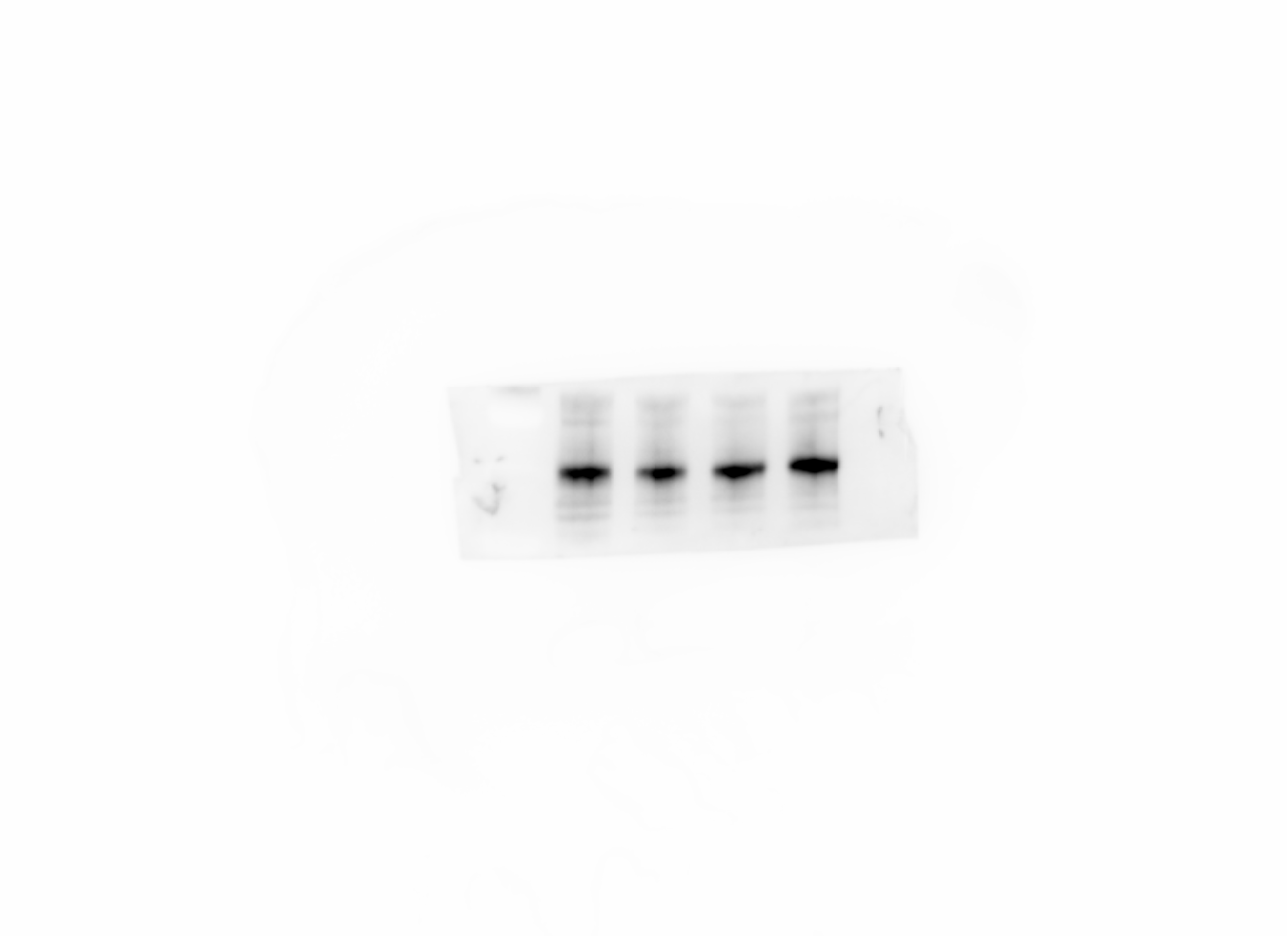

Supplement: Supplementary file 1 [file biomolecules-14-00237-s001.zip › biomolecules-2818843-supplementary/Original images of western Blot/original images/Figure 1A p-PAK1.tif]

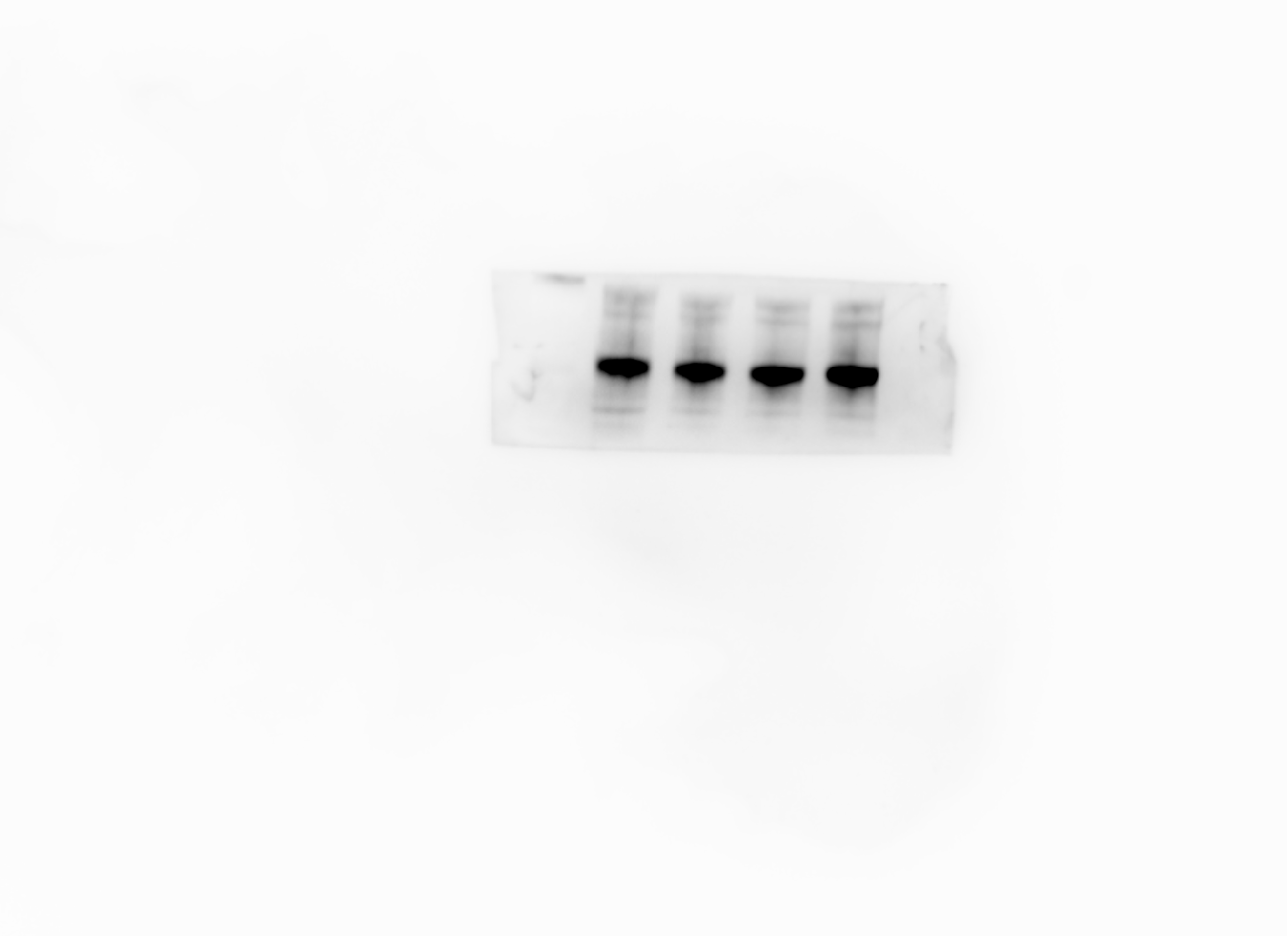

Supplement: Supplementary file 1 [file biomolecules-14-00237-s001.zip › biomolecules-2818843-supplementary/Original images of western Blot/original images/Figure 1A PAK1.tif]

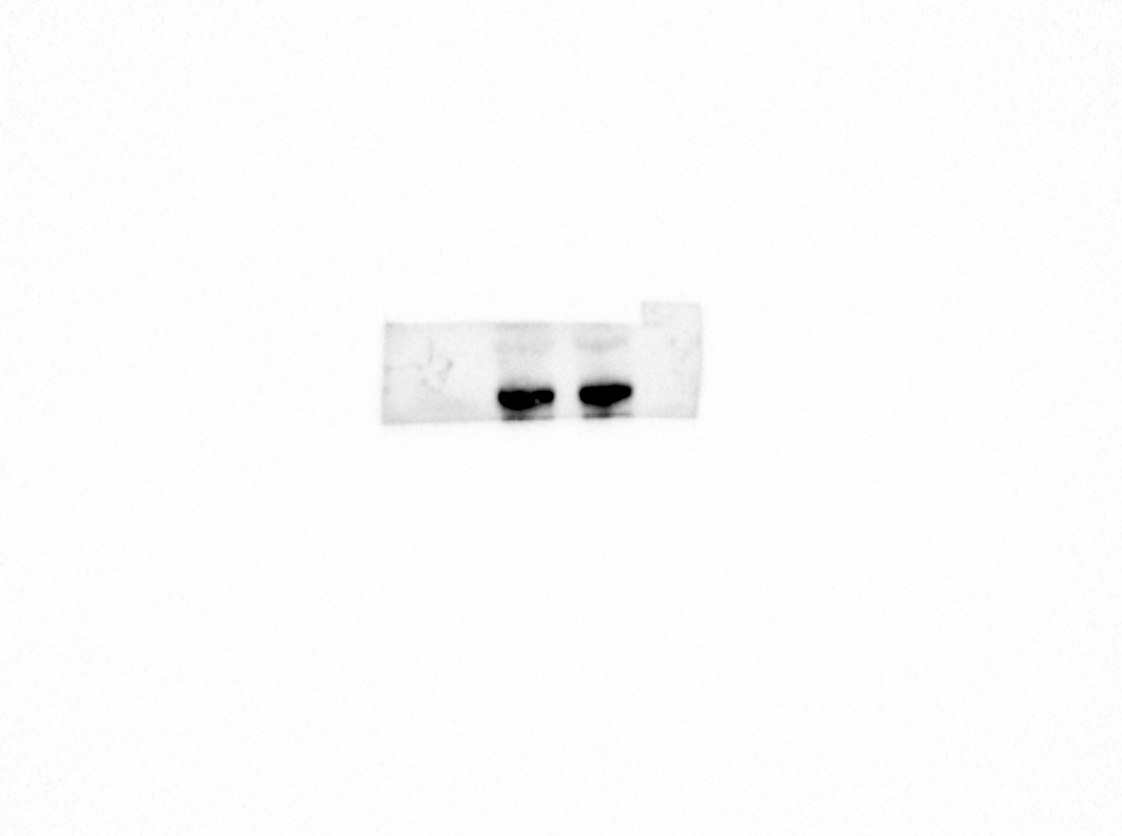

Supplement: Supplementary file 1 [file biomolecules-14-00237-s001.zip › biomolecules-2818843-supplementary/Original images of western Blot/original images/Figure 2F GAPDH.tif]

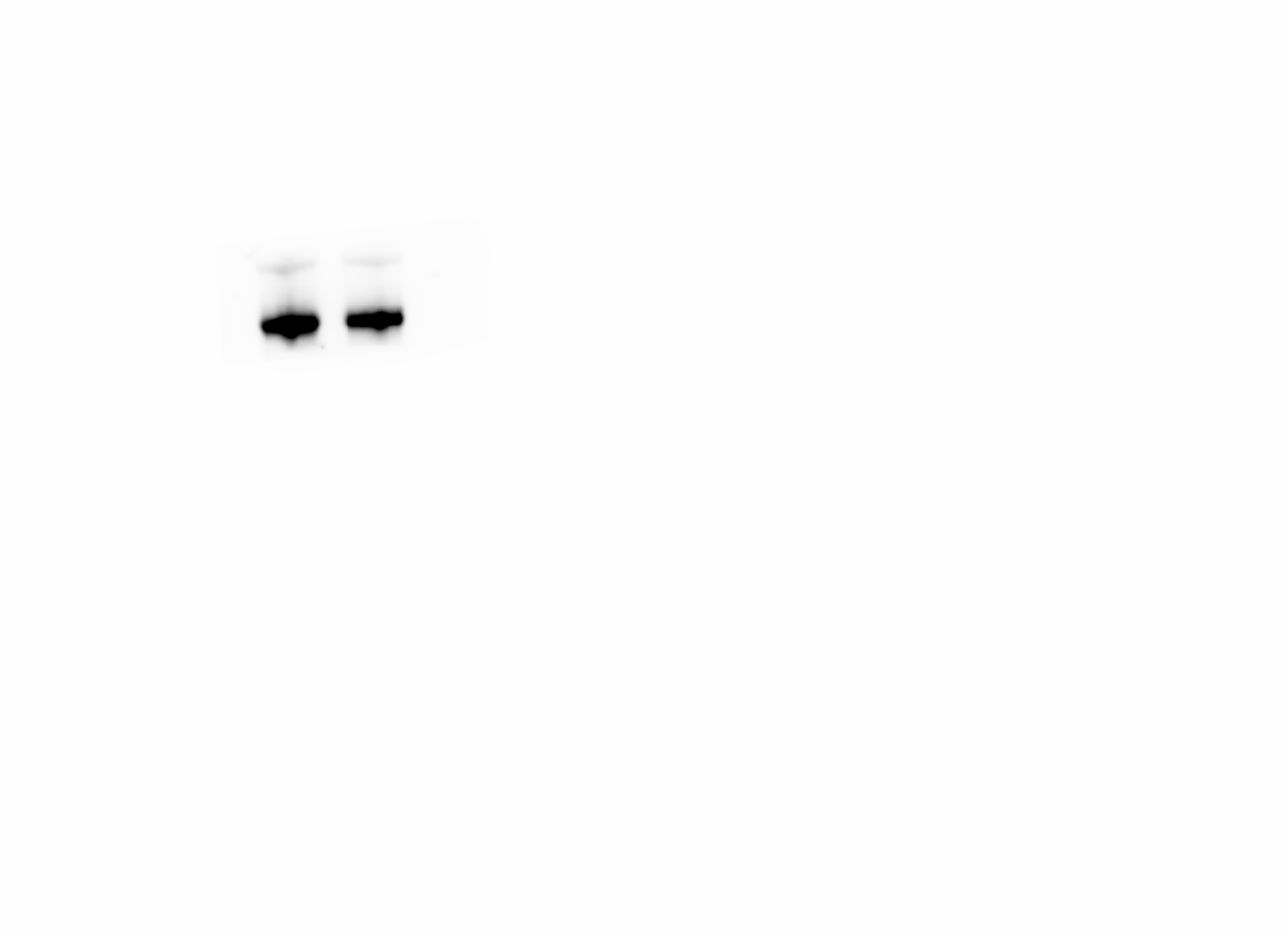

Supplement: Supplementary file 1 [file biomolecules-14-00237-s001.zip › biomolecules-2818843-supplementary/Original images of western Blot/original images/Figure 2F p-PAK1.tif]

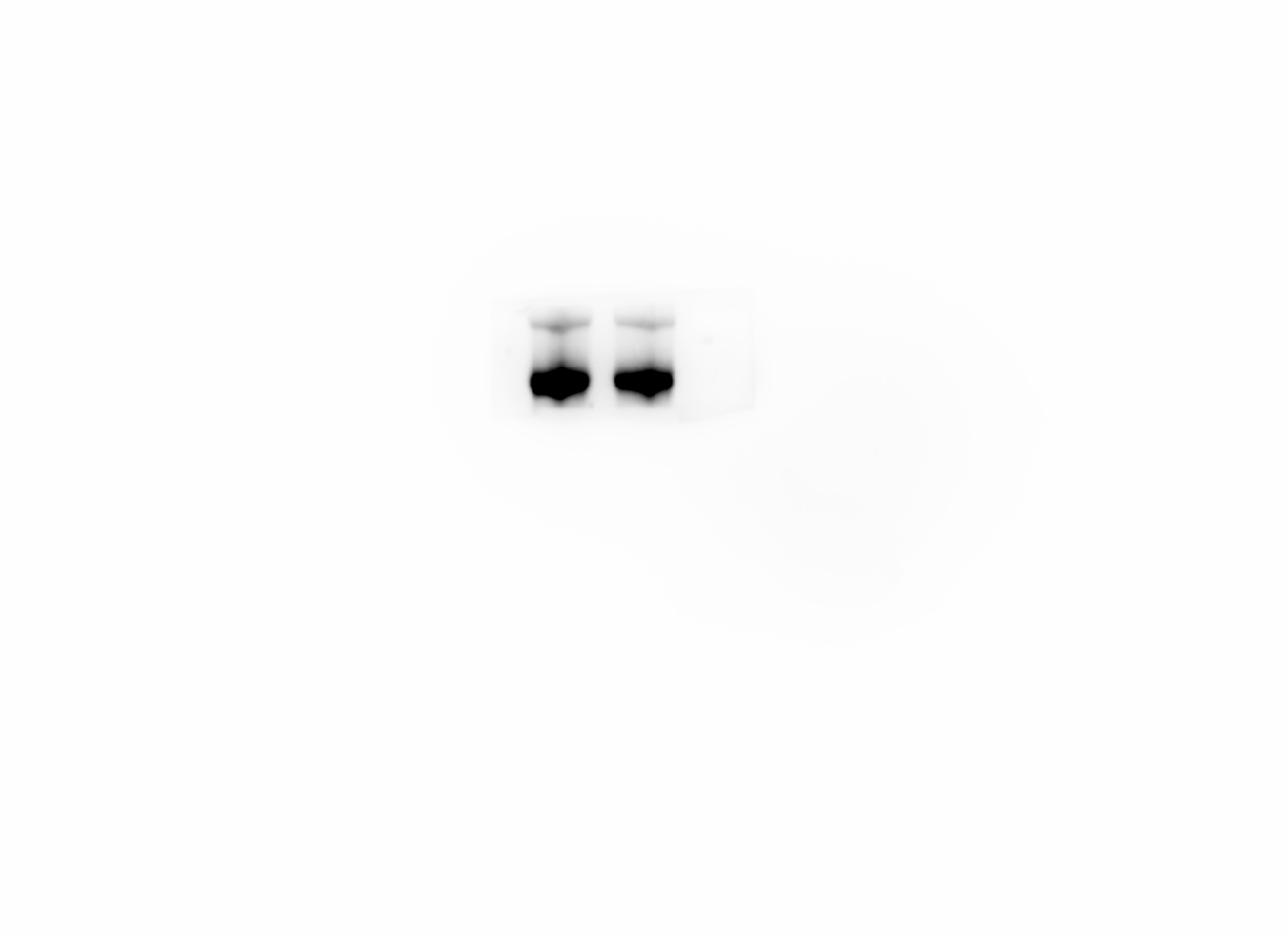

Supplement: Supplementary file 1 [file biomolecules-14-00237-s001.zip › biomolecules-2818843-supplementary/Original images of western Blot/original images/Figure 2F PAK1.tif]

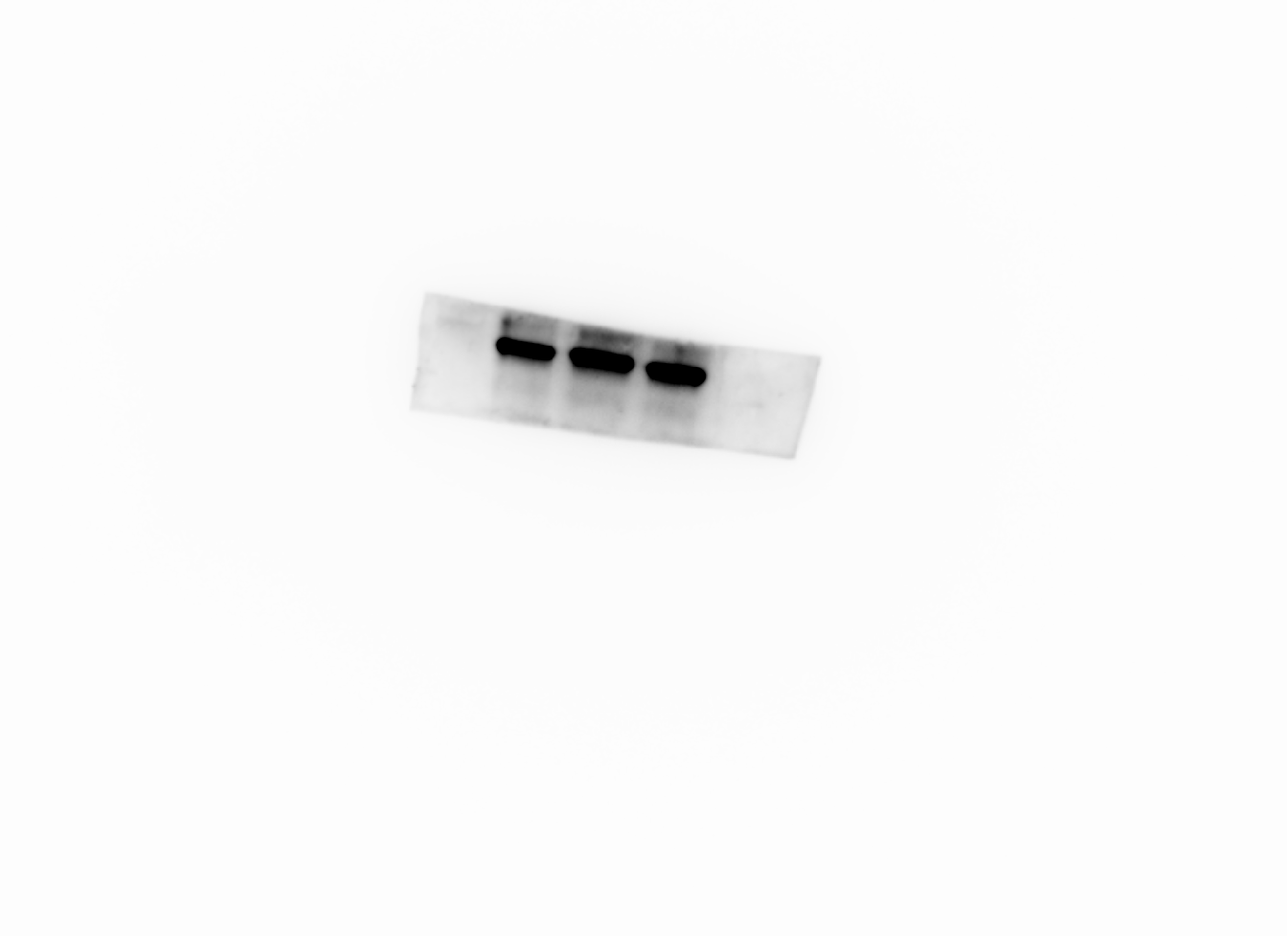

Supplement: Supplementary file 1 [file biomolecules-14-00237-s001.zip › biomolecules-2818843-supplementary/Original images of western Blot/original images/Figure 4F GAPDH.tif]

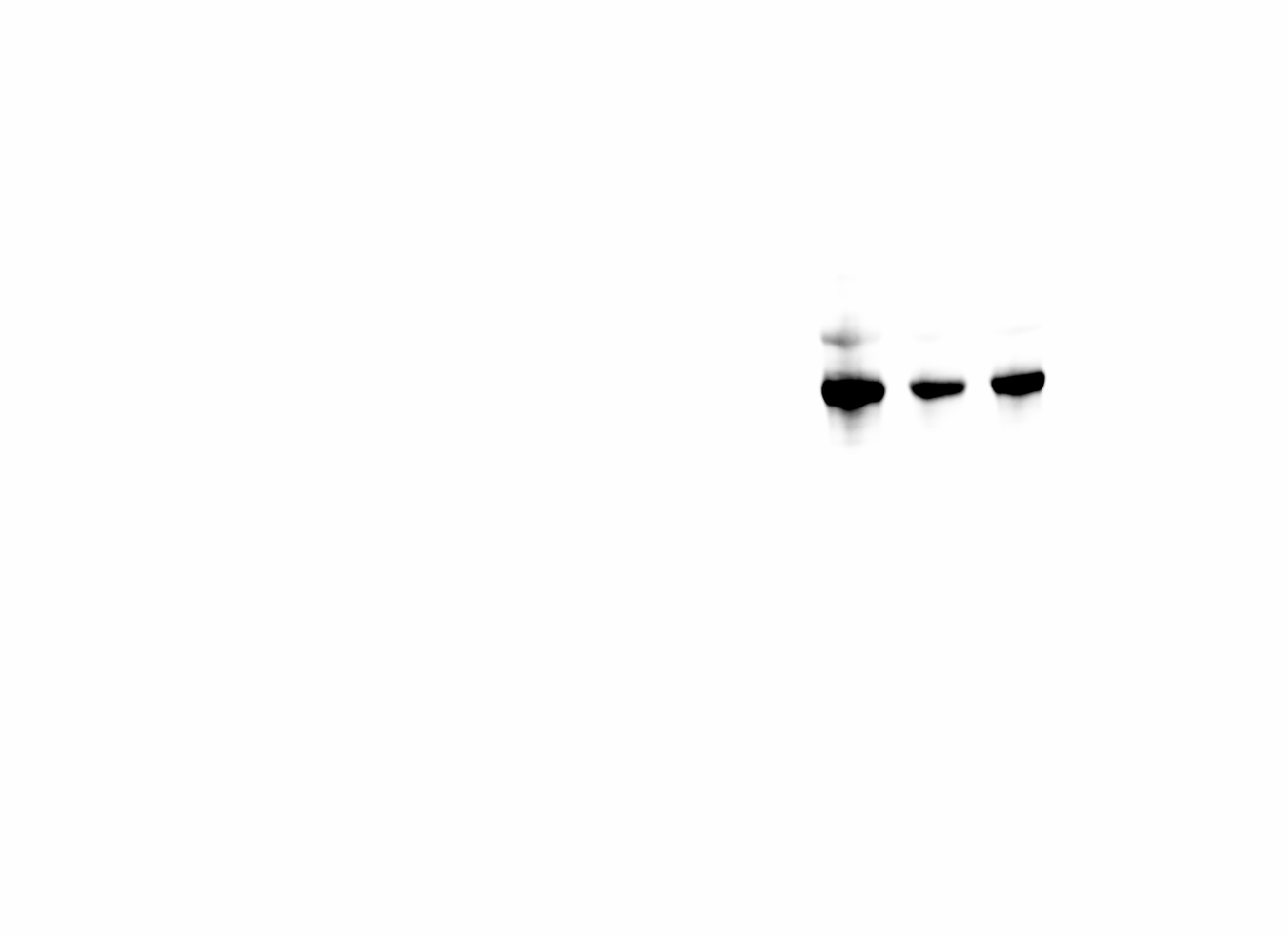

Supplement: Supplementary file 1 [file biomolecules-14-00237-s001.zip › biomolecules-2818843-supplementary/Original images of western Blot/original images/Figure 4F p-PAK1.tif]

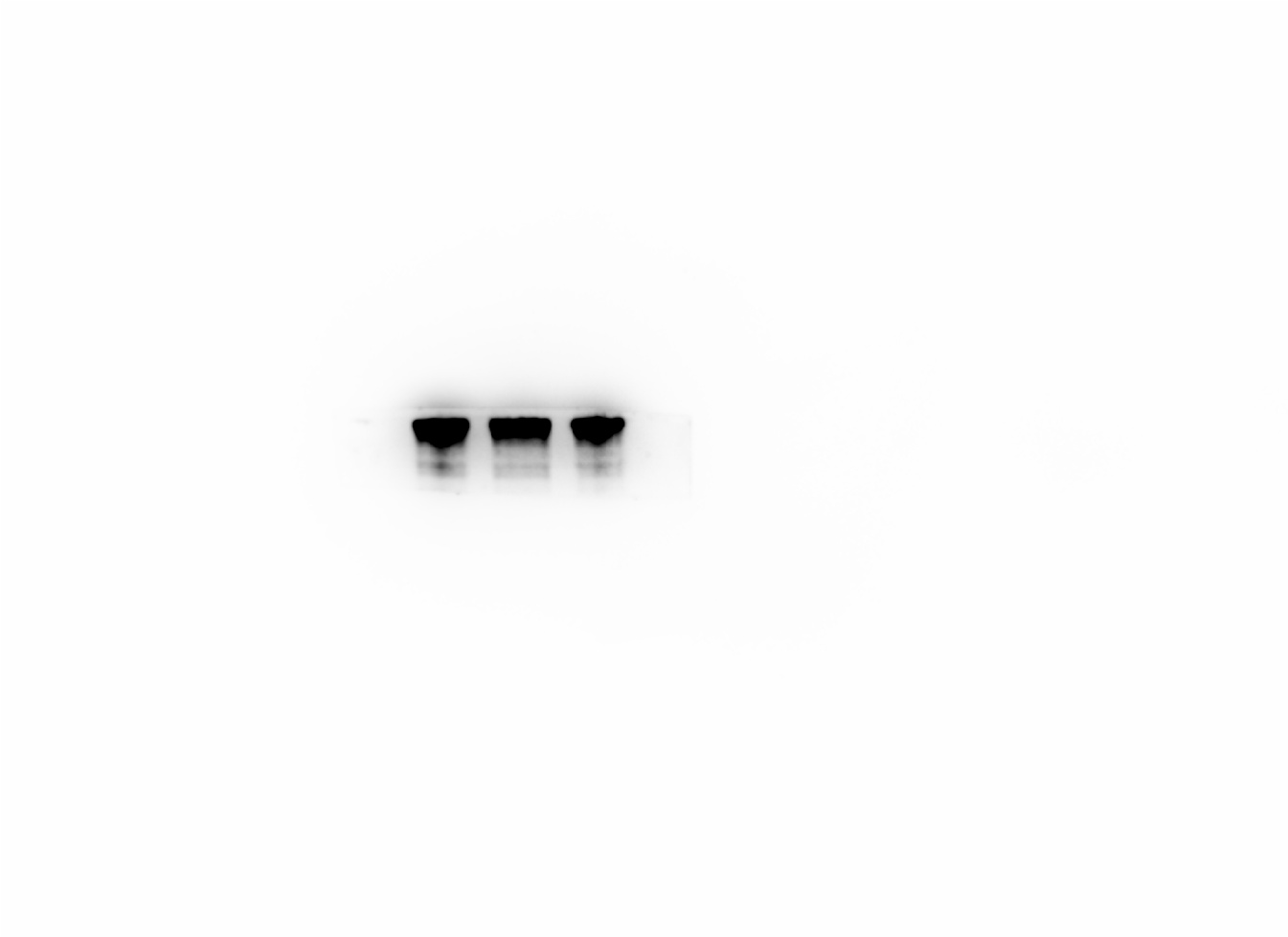

Supplement: Supplementary file 1 [file biomolecules-14-00237-s001.zip › biomolecules-2818843-supplementary/Original images of western Blot/original images/Figure 4F PAK1.tif]

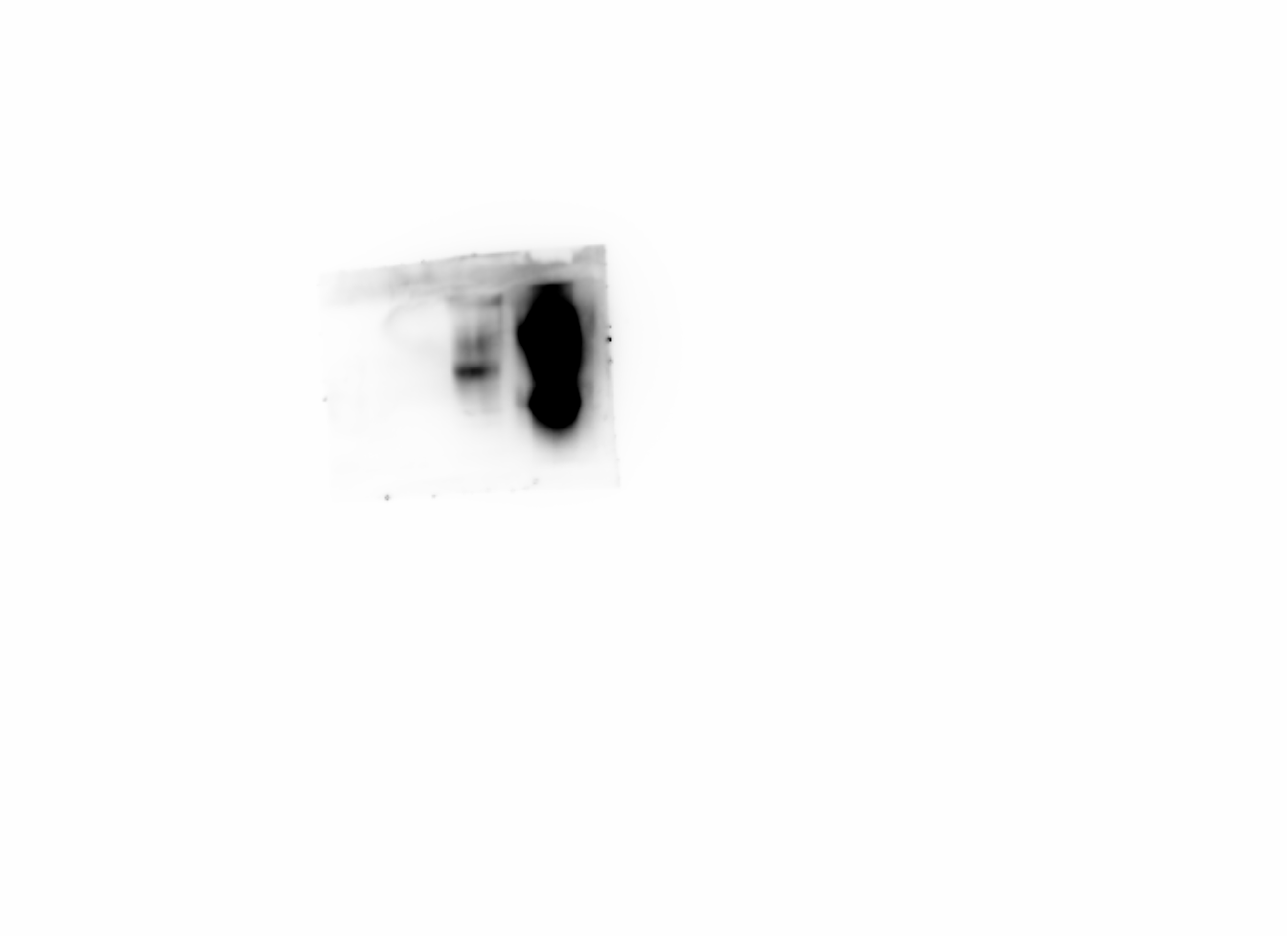

Supplement: Supplementary file 1 [file biomolecules-14-00237-s001.zip › biomolecules-2818843-supplementary/Original images of western Blot/original images/Figure 6A Aurora A.tif]

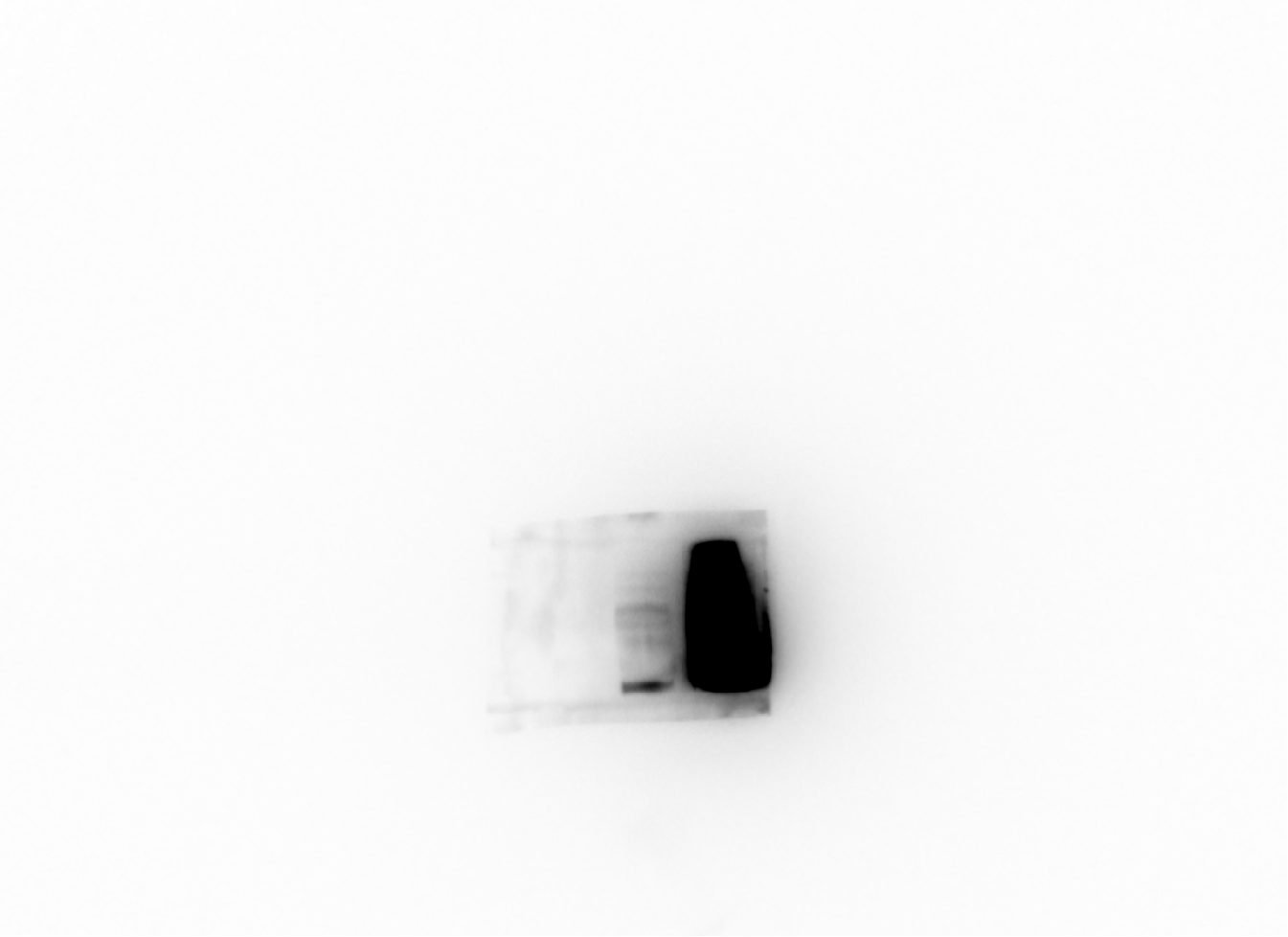

Supplement: Supplementary file 1 [file biomolecules-14-00237-s001.zip › biomolecules-2818843-supplementary/Original images of western Blot/original images/Figure 6A LIMK1.tif]

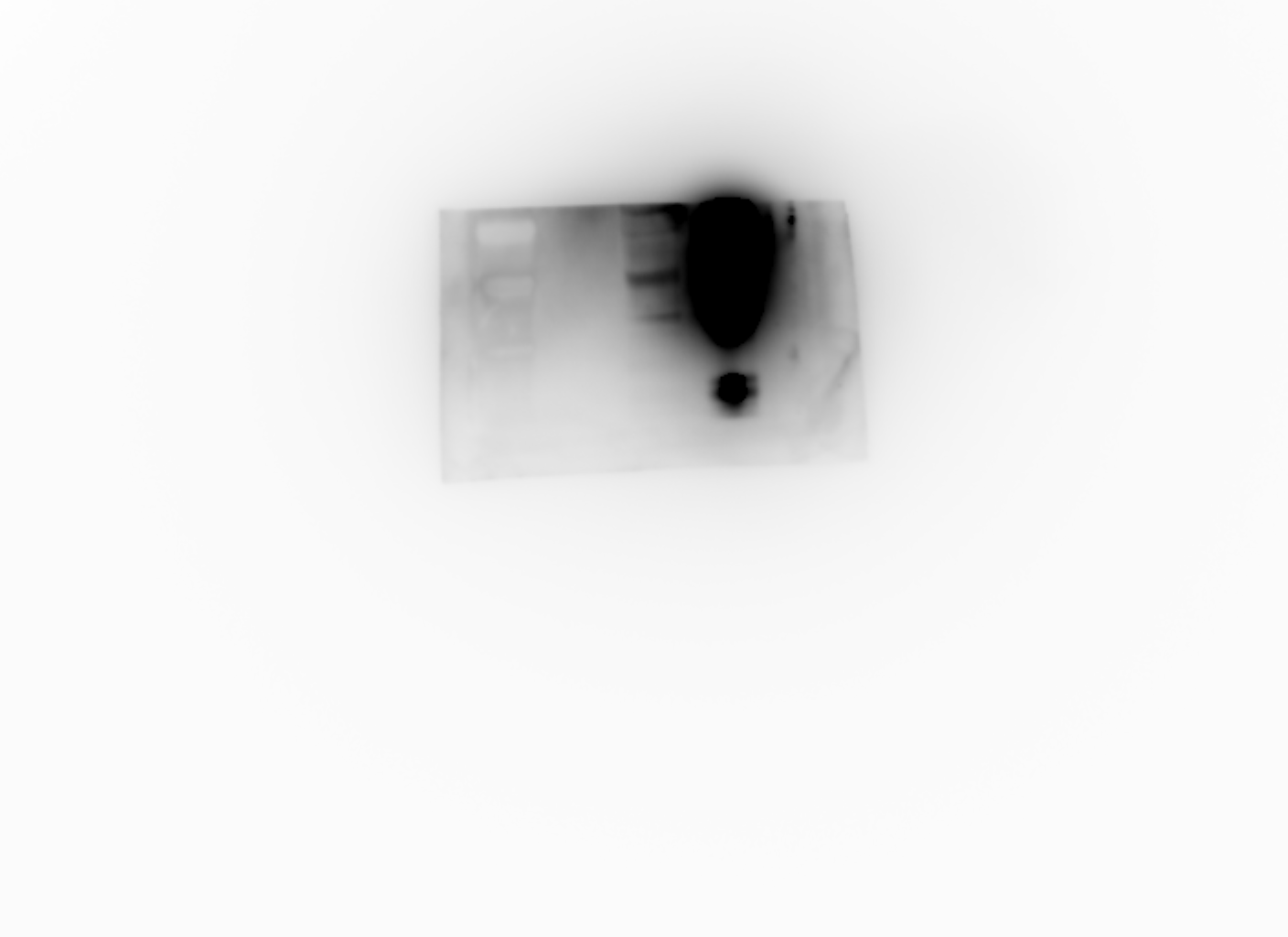

Supplement: Supplementary file 1 [file biomolecules-14-00237-s001.zip › biomolecules-2818843-supplementary/Original images of western Blot/original images/Figure 6A PAK1.tif]

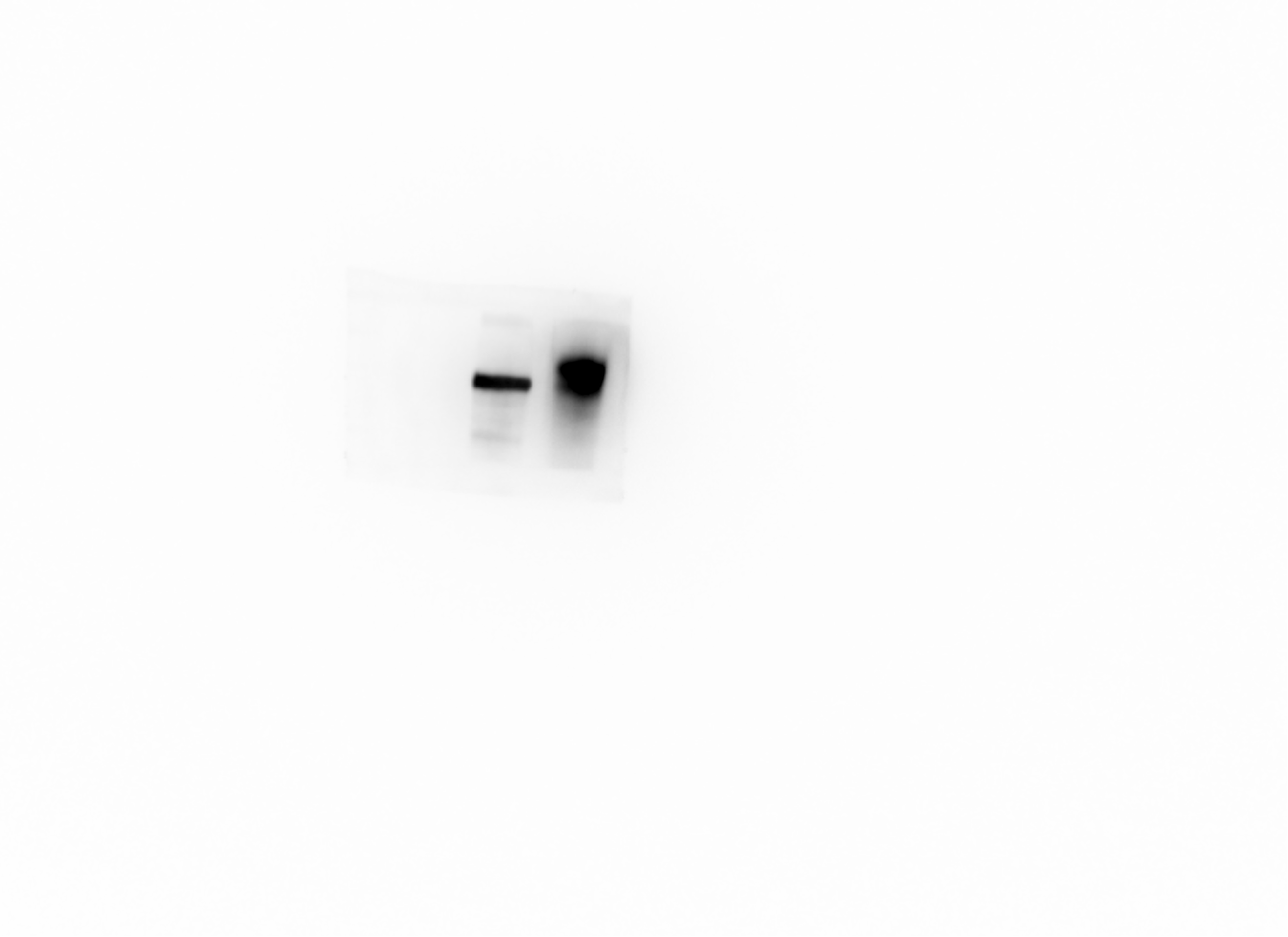

Supplement: Supplementary file 1 [file biomolecules-14-00237-s001.zip › biomolecules-2818843-supplementary/Original images of western Blot/original images/Figure 6A TACC3.tif]

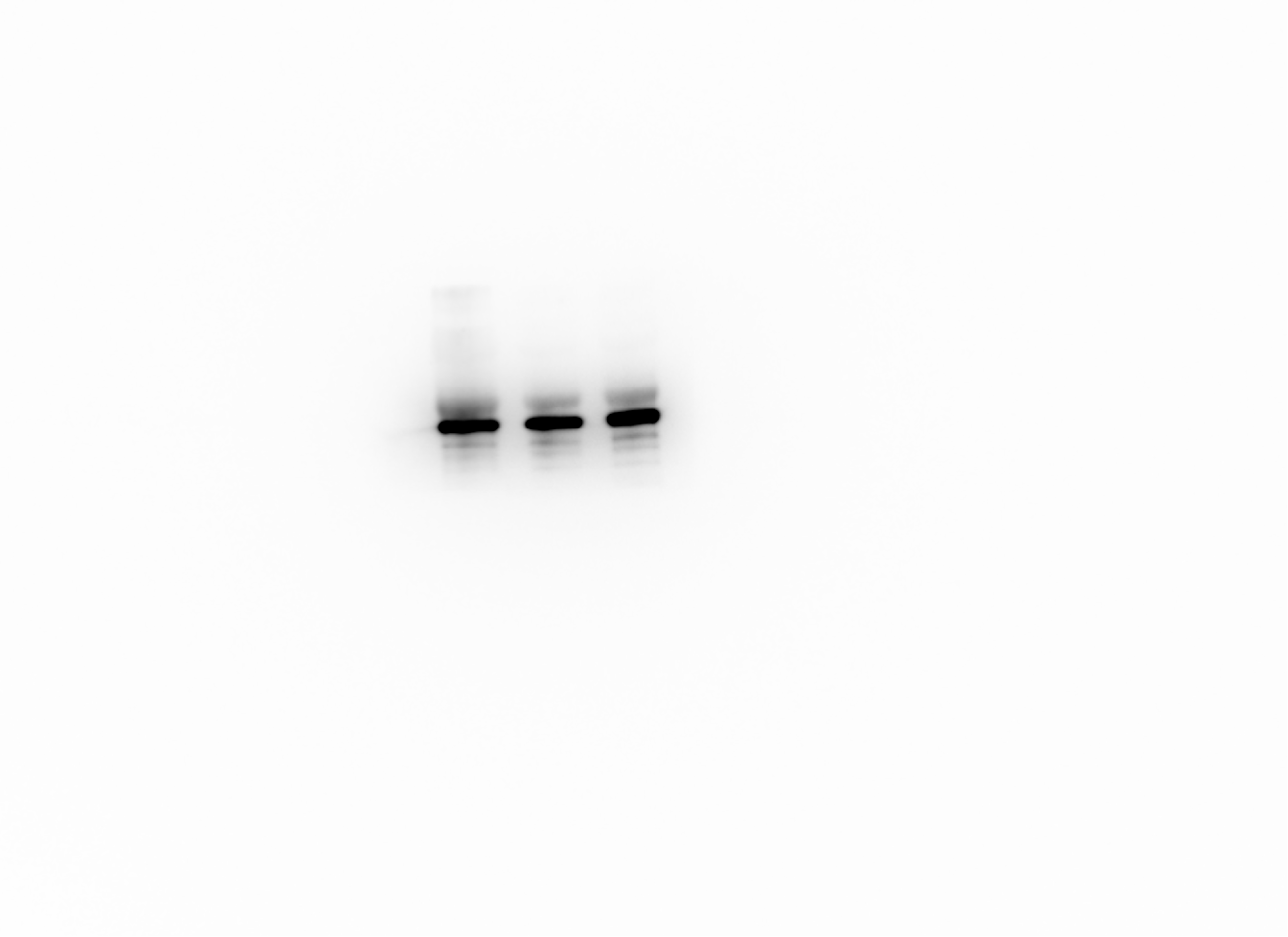

Supplement: Supplementary file 1 [file biomolecules-14-00237-s001.zip › biomolecules-2818843-supplementary/Original images of western Blot/original images/Figure 6B Aurora A.tif]

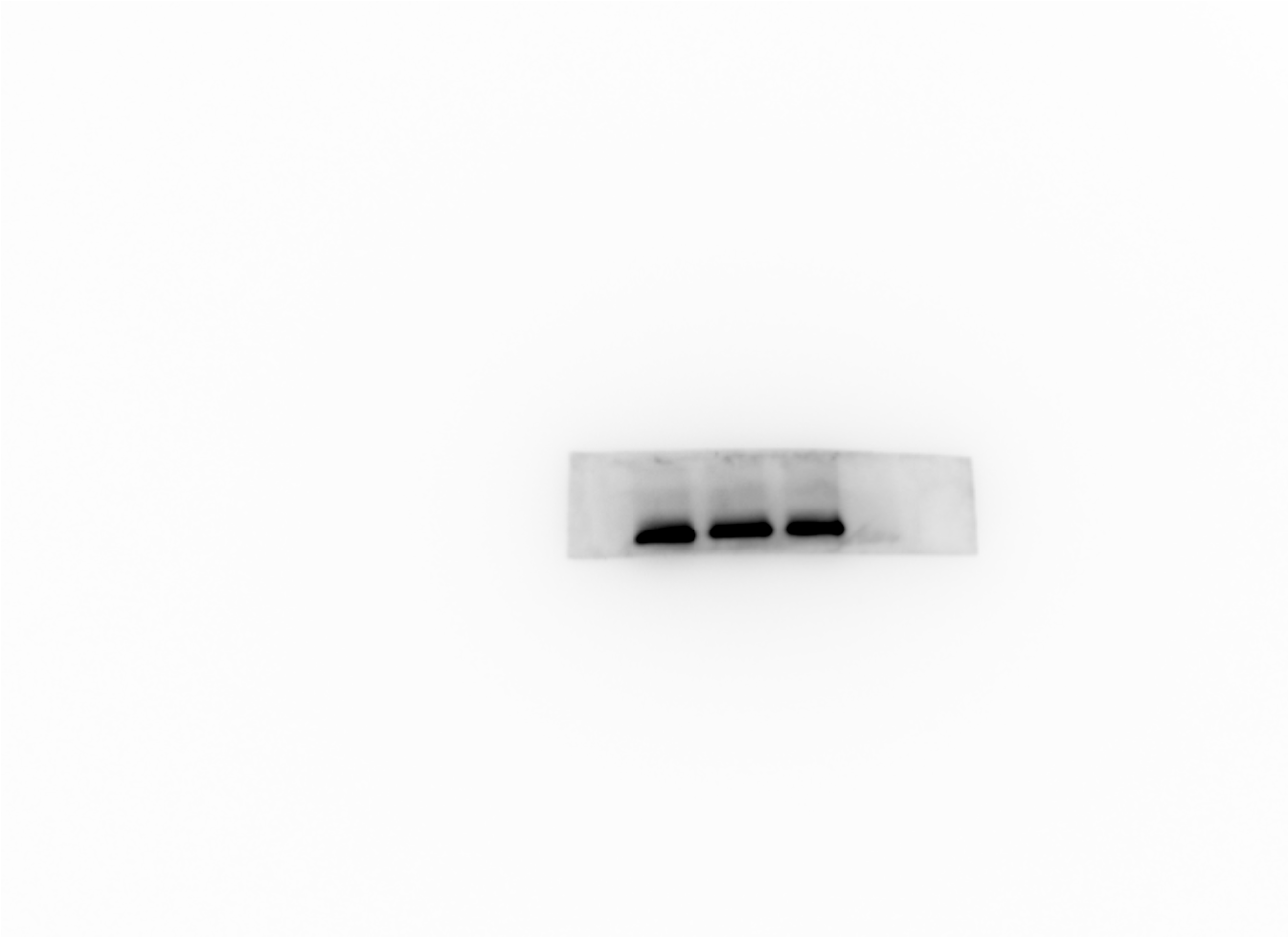

Supplement: Supplementary file 1 [file biomolecules-14-00237-s001.zip › biomolecules-2818843-supplementary/Original images of western Blot/original images/Figure 6B GAPDH.tif]

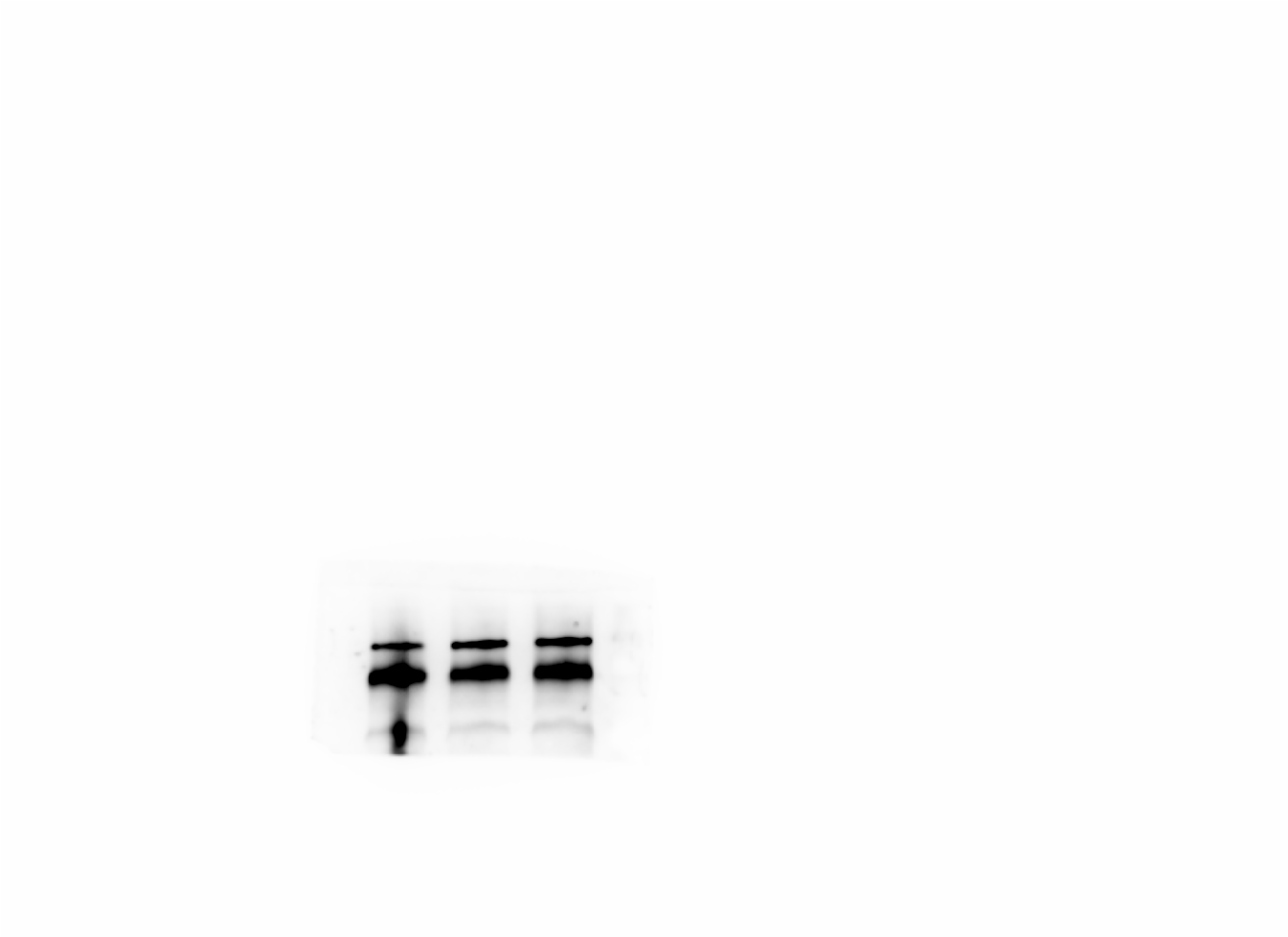

Supplement: Supplementary file 1 [file biomolecules-14-00237-s001.zip › biomolecules-2818843-supplementary/Original images of western Blot/original images/Figure 6B LIMK1.tif]

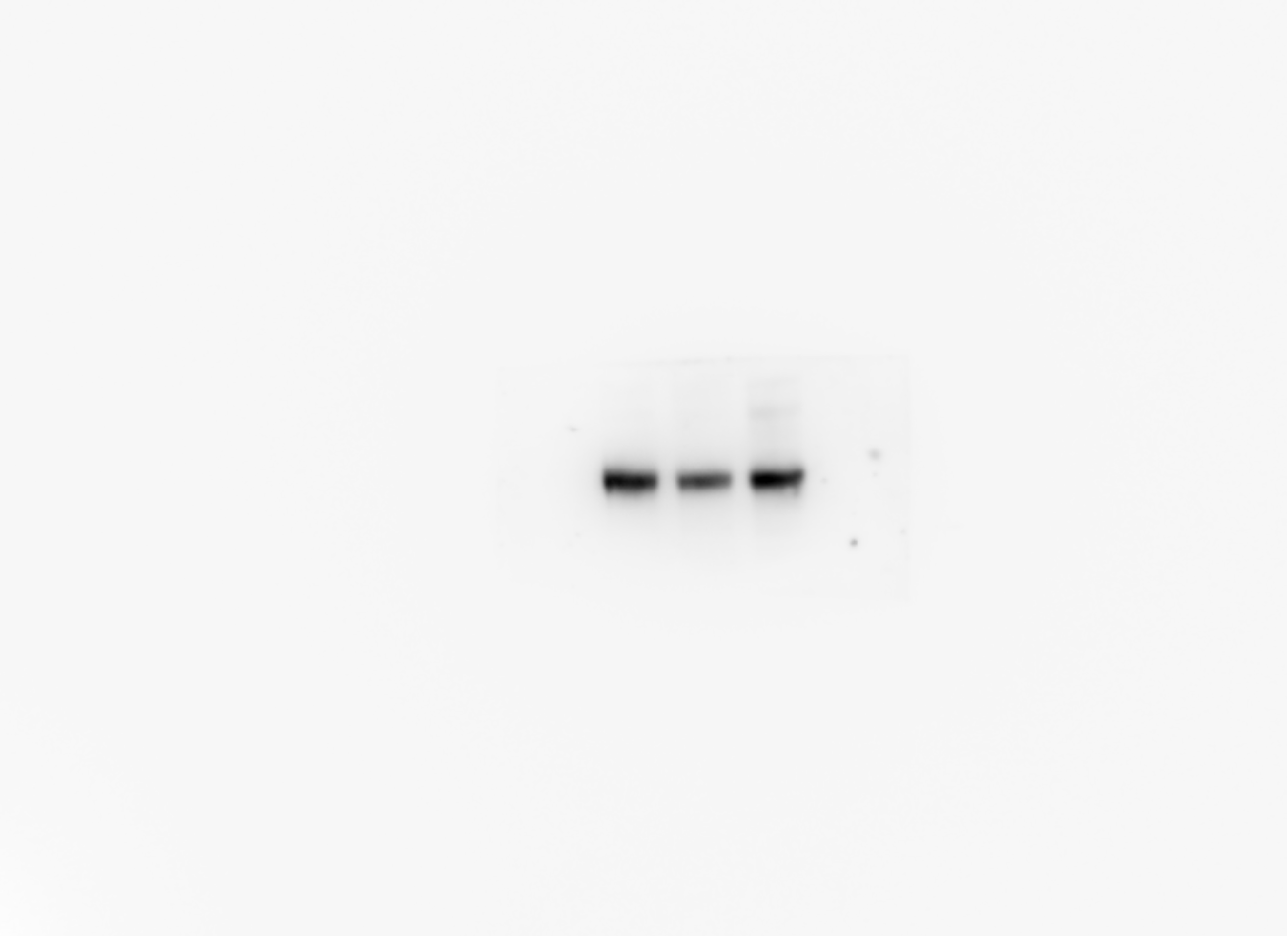

Supplement: Supplementary file 1 [file biomolecules-14-00237-s001.zip › biomolecules-2818843-supplementary/Original images of western Blot/original images/Figure 6B p-Aurora A.tif]

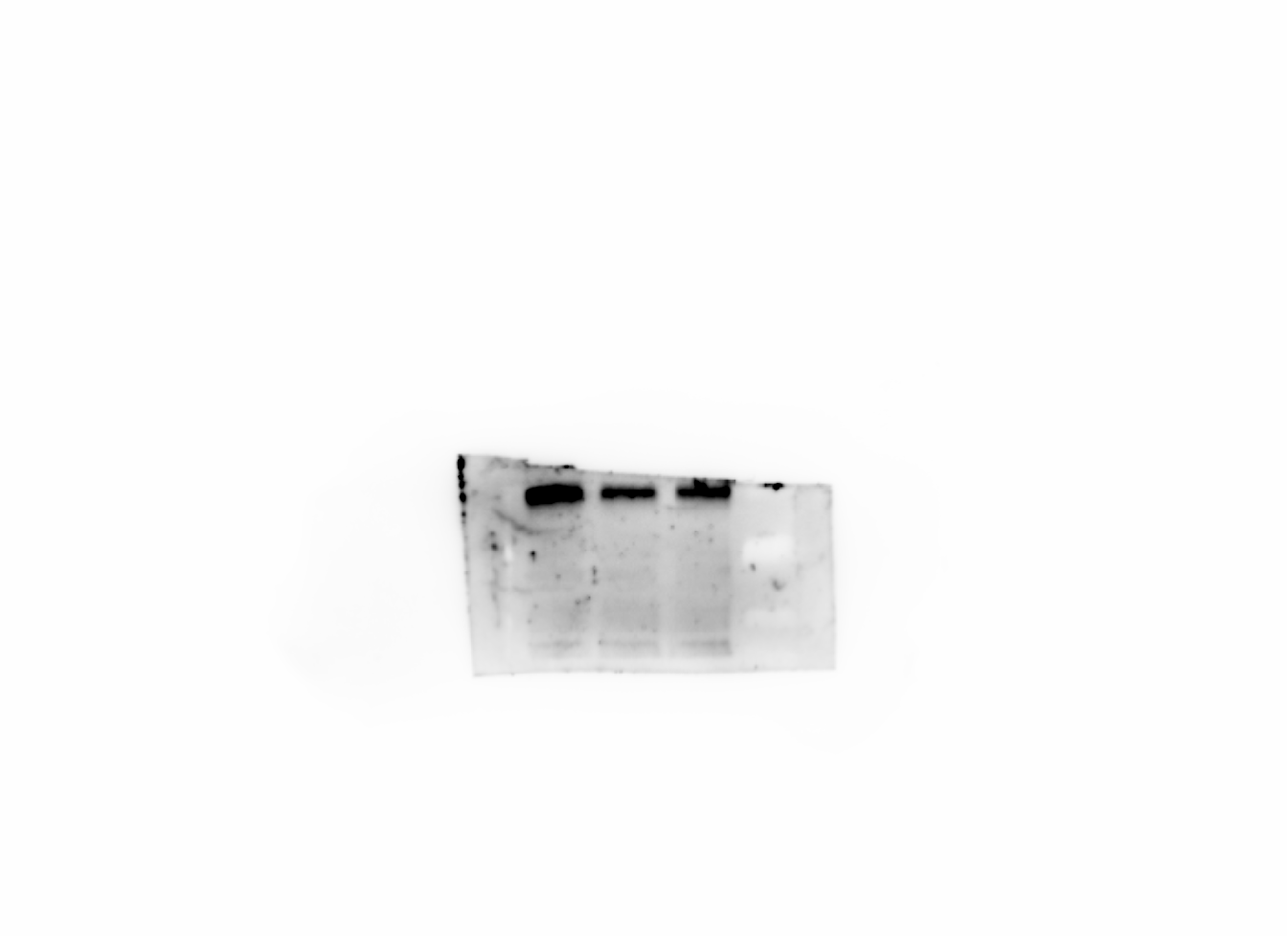

Supplement: Supplementary file 1 [file biomolecules-14-00237-s001.zip › biomolecules-2818843-supplementary/Original images of western Blot/original images/Figure 6B p-LIMK1.tif]

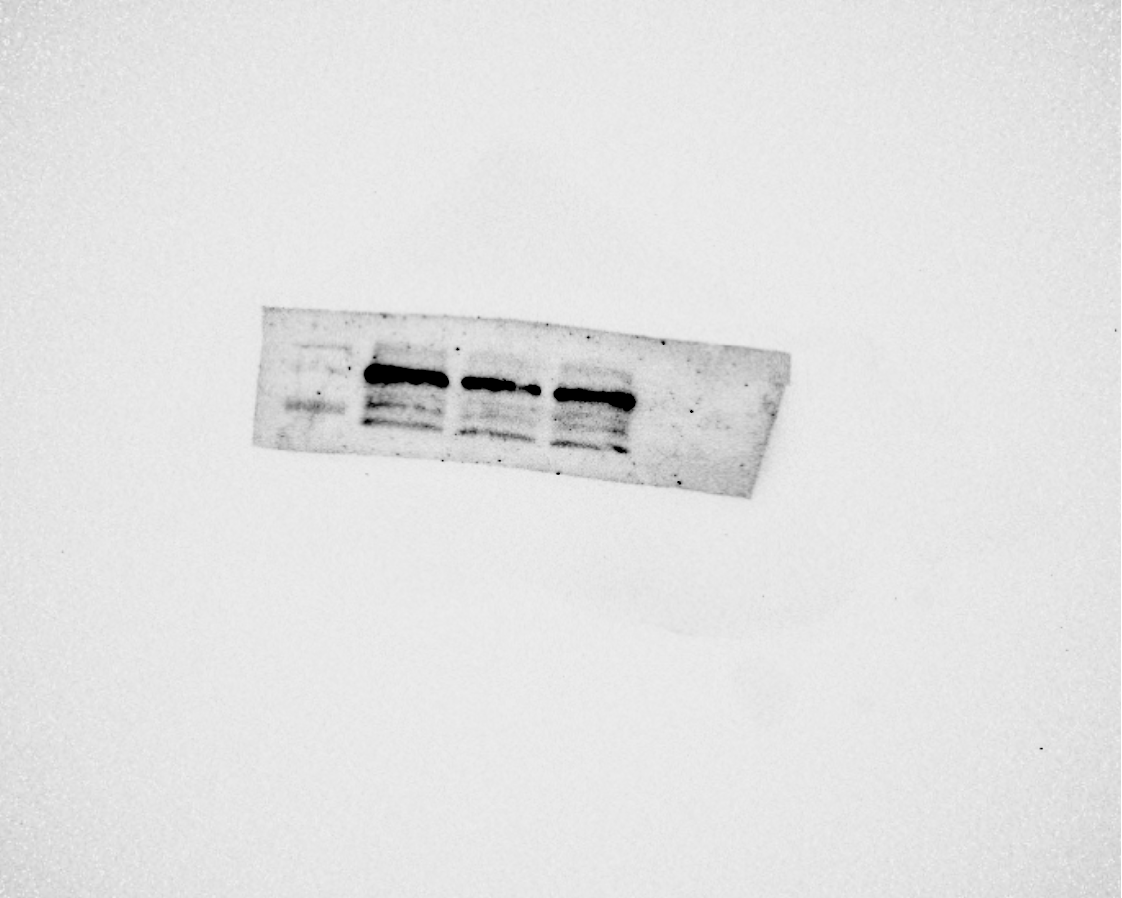

Supplement: Supplementary file 1 [file biomolecules-14-00237-s001.zip › biomolecules-2818843-supplementary/Original images of western Blot/original images/Figure 6B p-TACC3.tif]

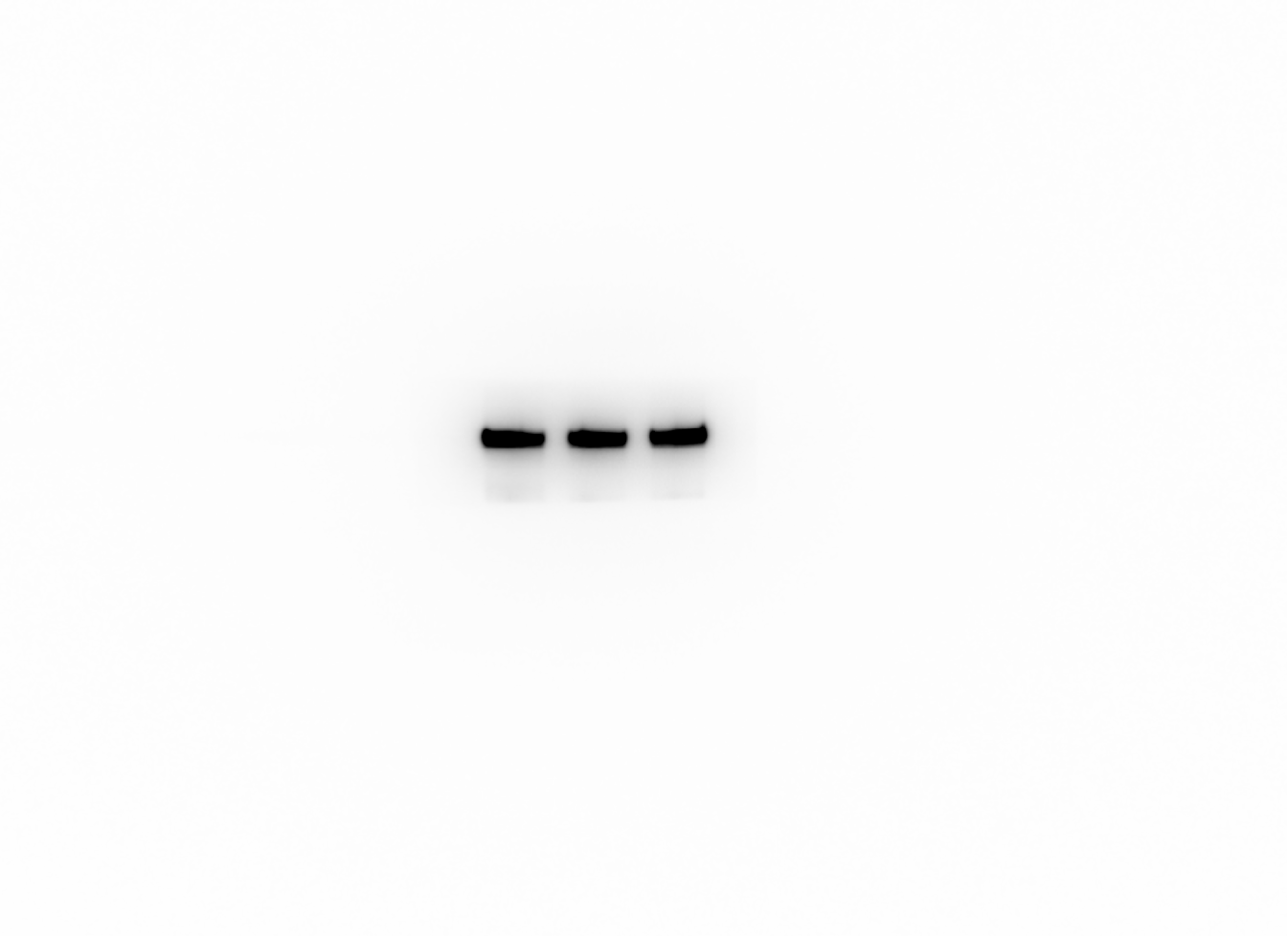

Supplement: Supplementary file 1 [file biomolecules-14-00237-s001.zip › biomolecules-2818843-supplementary/Original images of western Blot/original images/Figure 6B TACC3.tif]
